# Southern Dental Association

**Published:** 1887-12

**Authors:** 


(TO BE CONTINUED.)

---

### SOUTHERN DENTAL ASSOCIATION.

### NINETEENTH ANNUAL SESSION AT OLD POINT COMFORT, VA., 1887.

REPORTED FOR THE INDEPENDENT PRACTITIONER BY "MRS. M. W. J."

CONTINUED FROM PAGE 596.

Thursday was set aside for clinics, beginning at 8 A. M.

Dr. Younger, of California made two implantations—a lower central incisor, and a superior bicuspid.

Dr. A. E. Baldwin, of Chicago, demonstrated his method of immediate root-filling, with hot air treatment, devitalizing and filling the root of a superior bicuspid with gutta-percha. At a subsequent clinic, Dr. B. S. Byrnes, of Memphis, filled in the same tooth a compound crown and approximal cavity with gold, using soft foil in ribbons. He used his new hand-piece, of which the special feature is its ready adjustment to the engine. The force of the blow is given by a heavy hammer instead of a strong spring, and is regulated by a spring under the index finger, which increases the force at will, or checks it altogether.

Dr. Geo. Evans, of New York, gave several clinics with his seamless gold contour crowns, which are made in a mold and burnished down *à la Herbst.* In one case the roots were prepared and the crown made, ready for setting, in twenty-five minutes. Dr. Evans also exhibited specimens of his removable-plate bridges, gold crowns with removable porcelain fronts, and an instrument consisting of a solid silver bulb, with broach attached, for drying out root-canals and dentine.

Dr. W. N. Morrison, of St. Louis, filled, with gold wire, nerve-canals in tortuous roots.

Dr. Morgan, of Virginia, used Dr. D. B. Freeman's new double-loop spring clamps for the rubber-dam.

Dr. L. P. Dotterer, of South Carolina, placed on the roots of a superior bicuspid an all-gold crown, made at the chair.

Dr. H. A. Parr, treated and filled and crowned the abscessed roots of two central incisors, over which, without any treatment, a plate had been worn for seven years, the crown having been broken off by accident.

Dr. J. J. R. Patrick, of Belleville, Ills., exhibited his method of making crowns.

Dr. Genese, of Baltimore, gave several clinics in prosthetic dentistry with his new pinless teeth, using Rishel's Automatic Vulcanizer. He also demonstrated the practical value of his two new appliances, a syphon tongue-holder and a speculum and cheek distender.

Dr. J. G. Morey was on hand with his nerve crown drills. These drills have spiral cutting wings with a thin edge, which discharges all debris, and a triangular reamer with a non-cutting, cone-shaped point, which prevents piercing the foramen; the shank is also pliable, enabling the drill to conform to curvatures of roots.

In the S. S. White exhibit, Dr. A. T. Starr was stamping up crowns with their die-plate and hub-molds. They also show the vulcan lining for vulcanite plates, a combination of gold and silver sweated together and rolled and hammered into foil, giving a pure gold surface next the mucous membrane at a cost of not more than eighty cents per plate.

The Mann Vulcanizer; the Motor and Battery of the Detroit Motor Co.; the Shaw Dental Engine—an English patent with fixed upright, double-grooved pulley and duplex driving-spring; the Partz Acid Gravity Battery; Bing's Bridge Teeth; and a new slip-joint connection for coupling all hand-pieces, or the right-angle attachment, by a spring-catch, constitute the principal new items in their exhibit.

THURSDAY EVENING SESSION.

Dr. P. Beadles, of Danville, Va., read a paper, entitled "The Old and the New."

The discussion of Operative Dentistry was continued by Drs. Winckler, McKellops, Morgan, Storey and R. Finley Hunt.

A paper from Dr. Hilzim, of Mississippi, on "Mechanical Dentistry," was read by title. Dr. W. D. Dunlap, of Selma, Ala., read a paper, entitled "Dental Hygiene ; A Study that Belongs to the People."

Dr. Dunlap urged the importance of this much neglected branch, and especially the duty of the dental profession to instruct the people in the value of the teeth. They need specific instruction, and books should be prepared to be used in schools, inculcating correct hygienic principles. It should be the duty of teachers to require not only clean hands and faces, and well-brushed hair, but also clean teeth. If children were properly instructed, dental operators would be called in earlier with obvious advantage. The aid of legislatures and school-boards should be enlisted to make the study of this special branch of hygiene compulsory. He would also have it made the duty of certain practitioners to go into the field and instruct the people, through schools, the Y. M. C. A., etc., having them duly authorized and supported by State Dental Societies.

In this connection, Dr. Dunlap also read a communication from the State Society of Alabama on this subject, which was laid over for action next year.

This subject was discussed at considerable length, Drs. R. Finley Hunt, W. N. Morrison, J. Y. Crawford and others endorsing the author of the paper in his views of the great importance of the subject.

Dr. Hunt thought the trouble lay not so much in lack of cleanliness or care of the teeth, or improper diet, as in defective assimilation.

Dr. Morrison thought the teeth were suffering from lack of exercise, due to the use of artificially prepared foods requiring little mastication.

Dr. Hunt did not think we would hope to accomplish much in the present generation, but it was our duty to work for the future, the question of heredity entering largely into the matter.

Dr. W. H. Morgan, Dr. Storey, Dr. Hodgkins and Dr. W. H. Atkinson also took part in the discussion.

At the close of the discussion, Dr. A. W. Harlan, of Chicago, addressed the Association, on the part of a committee from the American Dental Association, on the subject of a joint meeting of the two Associations, and Drs. Knapp, Catching and E. S. Chisholm were appointed on the committee. The joint committee agreed upon the following report, which was adopted by the Association:

*First,* That the invitation of the American Dental Association to hold a meeting for social and scientific purposes be accepted.

*Second,* The two committees agree to recommend that Louisville, Ky., be the place, and the fourth Tuesday in August the date for holding such union meeting.

*Third,* That all details of arrangements for the consummation of this object be placed in the hands of the officers of the Associations, with power to act.

Prof. G. W. Hubbard, Dean of the Meharry College, of Tennessee, was introduced to the Association on Friday morning. He spoke at some length in behalf of the institution he represents, soliciting the coöperation of the Association in the professional and scientific education of colored youth, especially in the dental profession. He was listened to with great interest.

